# The Impact of Technology on Adolescent Sexual and Reproductive Needs

**DOI:** 10.3390/ijerph19148684

**Published:** 2022-07-17

**Authors:** Judith Bacchus Cornelius, Jaleesa Marshay Smoot

**Affiliations:** 1 School of Nursing, University of North Carolina at Charlotte, Charlotte, NC 28223, USA; 2 Program in Public Health: Epidemiology and Health Informatics and Analytics, University of North Carolina at Charlotte, Charlotte, NC 28223, USA; jsmoot3@uncc.edu

## 1. Introduction

The call for articles for the *International Journal of Environmental Research and Public Health* Special Issue “Using mobile technology to promote adolescent sexual and reproductive health (SRH)” was proposed to identify efforts to provide adolescent SRH services during the COVID-19 pandemic. During the pandemic, adolescents experienced limited access to SRH information, an increase in intimate partner violence, disruption in maternity care, and increased involvement in risky or exploitative work [1]. mHealth interventions were developed to provide adolescent SRH across a wide range of services. In our review, we found that the widest range of mHealth interventions for adolescent SRH during COVID-19 occurred in the United States, followed by Africa, Asia, Europe, and South America. Other articles in this issue will discuss additional adolescent SRH mHealth interventions developed to reduce challenges that adolescents faced during the pandemic. 

### 1.1. In the United States

In the United States, SRH telehealth interventions were found to increase self-efficacy with condom use [2] and were found feasible for screening for sexually transmitted infections [3]. With a focus on subgroups of adolescents, mHealth interventions targeted homeless adolescents [4], trans youth [5,6,7], and justice-involved youth [8]. This underscored the importance of mHealth interventions because numerous homeless and LGBTQ+ youth own a mobile phone, and many reported mental health issues, substance abuse, eating disorders, homelessness, risky sexual behaviors, and victimization during the pandemic. One mobile app, +Proud equipped families of LGBTQ+ youth to identify adaptive strategies to reduce the negative effects of stigma that many of their youth experienced [6]. When addressing pregnancy prevention for cisgender lesbian, gay, bisexual, and other sexual minority (LGB+) teens, Girl2Girl was a mHealth intervention found to be associated with higher rates of condom use and intentions to use birth control during the pandemic [7]. Justice-involved youth were especially vulnerable to risky sexual behaviors and mental health issues during the pandemic. Snow-hill et al [8] tested and adapted the PHAT-Life intervention with juvenile justice staff and found that this intervention could be sustained within the justice system setting.

With social media platforms, Snap Chat was found to be a promising way to distribute SRH educational materials during COVID. Of the 236 hospitalized teens who received a Snap Chat access code to SRH information, almost half accessed the site after being discharged from the hospital [9]. Friendship networks were also examined during the pandemic. The Grindr mobile phone app was found to be promising in getting adolescents diagnosed with syphilis and gonorrhea to identify others in their friendship networks who could be infected when compared to individual sexual contacts [10]. SIHLEplus was an intervention delivered via telemedicine to African American girls who lacked access to drug and sexual risk-taking prevention programs in urban areas. The intervention demonstrated usability and acceptability with drug prevention and in reducing sexual risk-taking behaviors during the pandemic [11]. Wilkinson et al. [12] found that telephone and video platforms were well suited to providing SRH care during the pandemic. They recommended that health care providers use an algorithm to address all possible SRH concerns during a pandemic.

### 1.2. In Africa

During the pandemic, the Inthistogether mHealth pregnancy prevention program was found to be feasible and acceptable with the delivery of 5–11 text messages per day for 8 weeks to Ugandan youth [13]. WhatsApp was used to deliver the mHealth peer supported intervention, Interactive transition support for 15 to 19 year-old adolescents living with perinatally acquired HIV (InTSHA) in South Africa [14]. The 4 Youth by Youth mHealth photo verification app for self-texting was found to be feasible by Nigerian youth [15]. Text messaging was recommended to provide family planning information to adolescent and young adult women in Sierra Leone [16]. In Zambia, the Insake mobile phone app provided support to pregnant women living with HIV [17]. An average of 169 text messages were sent per user.

### 1.3. In Asia

In India, the artificial intelligence app, SnehAl Chatbot, was found to be innovative in engaging hard-to-reach populations with sexual health topics [18]. During this time, the HIV Info Corner app was developed for Indonesian youth [19]. After further evaluation, the HIV Info Corner app was found to have major and minor problems that were addressed.

### 1.4. In Europe and South America

In Italy, the need for abortion services increased during the pandemic. Brandell et al [20] found that telemedicine was an alternative way to provide women access to abortion services. In Brazil, telemedicine services increased with the launch of the PrEP 1519 demonstration program [21]. Participants were recruited on social media sites, hook-up apps, word of mouth, and using the Amanda Selfie AI chatbot. Social and mental health services were added to allow for more comprehensive health care [21]. 

## 2. Conclusions

In many countries, there was a proliferation of mHealth technology to ensure that adolescents received SRH, mental health, and drug prevention information during the pandemic. The authors in our special issue will highlight innovative strategies that they used to meet adolescents’ SRH needs during COVID-19. We believe that these articles will challenge traditional paradigms about health promotion during a pandemic. 
